# High levels of cerebrospinal fluid soluble triggering receptor expressed on myeloid cells 2 might be a biomarker of activity in pediatric patients with MOG-AD

**DOI:** 10.3389/fped.2022.908527

**Published:** 2022-10-13

**Authors:** Anna Zhou, Weihua Zhang, Changhong Ren, Ji Zhou, Haoxiao Chang, Xiaotun Ren

**Affiliations:** ^1^Department of Neurology, Beijing Children’s Hospital, Capital Medical University, National Center for Children’s Health, Beijing, China; ^2^China National Clinical Research Center for Neurological Disease, Beijing Tiantan Hospital, Capital Medical University, Beijing, China

**Keywords:** MOG-AD, CSF, sTREM2, microglia, autoimmune diseases

## Abstract

Myelin oligodendrocyte glycoprotein antibody disease (MOG-AD) is characterized by its monophasic or relapsing course and inflammatory demyelinating condition which is unable to be classified in typical multiple sclerosis (MS) or other known neuroinflammatory conditions. In the condition of neuroinflammatory, activated microglia are essential for demyelination. The secreted ectodomain of soluble triggering receptor expressed on myeloid cells 2 (sTREM2), expressed by microglial cells, is associated with abnormal biological pathways. It is known that the cerebrospinal fluid (CSF) sTREM2 concentration is much higher in neuroinflammatory and neurodegeneration diseases. However, the role of activated microglia has not been reported in MOG-AD pediatric patients. For the first time, the increased CSF and serum sTREM2 concentration in pediatric patients with MOG-AD is investigated in this work, showing evidence of microglia activation in MOG-AD. CSF sTREM2 levels significantly correlated with clinical inflammatory indexes and adapted modified Rankin Scale score, indicating the potential value of sTREM2 as a severity biomarker.

## Introduction

It is well known that myelin oligodendrocyte glycoprotein antibody-associated disease (MOG-AD), an antibody-mediated autoimmune demyelinating disorder in the central nervous system, is still a challenging nosologically entity. The frequency of anti-MOG antibody seropositivity is high in children with acute demyelinating syndrome presentations (1). In MOG-antibody-positive children, the median age at presentation is 6 years with same-gender bias as adults (2, 3). The clinical manifestation includes various phenotypes: from (mostly recurrent) optic neuritis (ON), *via* transverse myelitis (TM), and longitudinally extensive transverse myelitis (LETM) to acute disseminated encephalomyelitis (ADEM) and cortical encephalitis (4). This mono- or multiphasic course of neurological deficits makes MOG-AD difficult to diagnose with other known neuroinflammatory illnesses. With the new-generation cell-based assays, MOG-IgG demonstrated a robust association with the immunopathogenesis in MOG-AD (5). Even though testing serum MOG antibody IgG made the distinguishment of MOG-AD from typical multiple sclerosis (MS) or other common forms of neuroinflammation more specific and faster, the role of MOG antibody IgG in MOG-AD remains unclear (6).

On the other hand, in neuroinflammation, demyelination is associated with activated microglia (7). Triggering receptor expressed on myeloid cell-2 (TREM-2), a membrane-bound immune receptor, expressed on the differentiated and activated macrophages. Specifically, it is only expressed in peripheral macrophages and central microglia (8). The expression of TREM-2 is upregulated on activated microglia, modulating the biological function of phagocytosis (8, 9), survival (10), chemotaxis, and response to neuronal injury (11). The soluble triggering receptor expressed on myeloid cells 2 (sTREM2) is measurable by cleavage of the ectodomain, as the TREM-2 releases sTREM2 in cerebrospinal fluid (CSF) and blood (8). By this means, it is possible for the sTREM2 to become a marker to describe neuroinflammatory conditions (12).

The objective of this work is to explore the relationship between the pediatric MOG-AD and the sTREM2 levels in serum and CSF. In the meantime, the correlation between the CSF sTREM2 levels and the clinical inflammatory indexes is investigated by analyzing the sTREM2 in CSF samples from the pediatric MOG-AD group and control group.

## Method

### Patient selection and biofluid collection

The study included 19 control patients and 19 pediatric MOG-AD patients. Nineteen patients were selected from non-neuroinflammatory diseases as control groups, including two cases of mitochondrial disorders, one case of dizziness, one case of febrile convulsion, one case of migraine, three cases of somatization disorder, one case of acute concomitant strabismus, one case of intracranial hypertension, and nine cases of functional headache. Clinical data were retrospectively collected from hospitalized patients diagnosed with MOG-AD at the neurology department of Beijing Children’s Hospital between June 2018 and August 2021. The data was acquired from The FUTang Updating medical REcords (FUTURE) database. More details and the process of data cleaning have been presented in a previous publication (13). We recorded the medical history, neurological symptoms and signs, and biofluid laboratory testing results. According to the guidelines of MOG-AD in the USA, Europe, and related literature (4, 14, 15), the criteria for the diagnosis of MOG-AD in our study include (1) ON; (2) myelitis; (3) ADEM; (4) fundoscopy; 5) the onset of disease from 4 days to 4 weeks after vaccination, co-existing teratoma or NMDAR encephalitis; and (6) anti-MOG antibody testing.

All patients with MOG-AD were in the acute phase, and the samples were collected prior to treatment. CSF and serum samples were immediately centrifuged and the supernatant was collected and stored at −80°C until the time of the ELISA assays.

This study was approved by the Ethics Committee of Beijing Children’s Hospital Affiliated to Capital Medical University, Beijing, People’s Republic of China, and written informed consent was obtained from all selected participants and their parents.

### ELISA for detection of soluble triggering receptor expressed on myeloid cells 2

The CSF and serum analyses on sTREM2 were performed using a commercially available ELISA assay from Abcam (Abcam, USA, ab224881) according to the manufacturer. Neat CSF samples and serum samples were analyzed on the same day using assays from the same lot to avoid inter-lot variations. CSF samples and serum samples from the control group were evenly distributed on the plates.

### Statistical analysis

Data were analyzed by SPSS software (IBM SPSS 22.0 version) and Prism (GraphPad software 8.0 version). As a small sample quantity, the distribution of CSF and serum level of sTREM2 obey normal (Shapiro–Wilk test, *p* > 0.05), both in pediatric MOG-AD and control groups. The distribution of CSF WBC count and CSF protein concentration were not normal (Shapiro–Wilk test, *p* < 0.05) in the pediatric MOG-AD group, but were normal (Shapiro–Wilk test, *p* > 0.05) in the control groups. Differences between pediatric MOG-AD and control groups were assessed with an independent two-sample *t*-test when the data were parametric. Correlation coefficients were calculated using Pearson’s two-tailed correlation test when all variables satisfy normal distribution. Spearman’s two-tailed correlation test was employed for correlation coefficients when any variables did not satisfy normal distribution. Receiver operating characteristic (ROC) curves were performed to assess the diagnostic value.

## Results

### Patients’ characteristics

The clinical characteristics and concentrations of biomarkers for the enrolled patients are shown in Table 1. As described in Table 1, a total of 19 MOG-AD pediatric patients (10 male and 9 female) and 19 control patients (10 male and 9 female) were included. All patients from the control group were selected from non-neuroinflammatory diseases. Informed consent was obtained from all participants and their parents. The study was approved by the local ethical board of Beijing Children’s Hospital.

**TABLE 1:** Clinical and demographic characteristics of participants.

Subject details	Con	MOG-AD
Age, mean ± SD, m	114.47 ± 44.265	76.00 ± 36.741
Sex, no. (%)		
Male	10 (52.6%)	10 (52.6%)
Female	9 (47.4%)	9 (47.4%)
**Adapted modified Rankin Scale (mRS) score**		
Score 0, no symptoms	−	0
Score 1, non-disabling symptoms	−	1 (5.7%)
Score 2, minor symptoms	−	3 (15.6%)
Score 3, moderate symptoms	−	7 (36.8%)
Score 4, moderately severe symptoms	−	5 (26.3%)
Score 5, severely disabled	−	3 (15.6%)
Score 6, dead	−	0
Expanded Disability Status Scale (EDSS) score	−	2 (3−4)
CSF WBC count (× 10^6^/L), median (IQR)	7 (4−9)	38 (2−65)
CSF protein concentration (mg/L), median (IQR)	251 (198−580)	244 (195−379)
Serum sTREM2^a^ (ng/ml), median (IQR)	10.84 (9.28−11.59)	14.42 (11.55−16.47)**
CSF sTREM2^a^ (ng/ml), median (IQR)	13.69 (9.04−17.53)	32.63 (16.62−42.72)***
Clinical picture		
Optic neuritis	−	2 (10.5%)
ADEM	−	15 (79%)
Others	−	2 (10.5%)
Overlap NMDA	−	3

Data expressed as mean ± SD or median ± SD (IQR) as appropriate.

MOG-AD, myelin oligodendrocyte glycoprotein-associated disease; m, month; WBC, white blood cell; CSF, cerebrospinal fluid; sTREM2, soluble triggering receptor expressed on myeloid cell 2; ADEM, acute disseminated encephalomyelitis.

^a^Differences in CSF sTREM2 levels and serum sTREM2 levels between control groups and MOG-AD pediatric groups were assessed by an independent two-sample *t*-test.

**p < 0.01, ***p < 0.001.

### Cerebrospinal fluid and serum soluble triggering receptor expressed on myeloid cells 2 levels are higher in MOG-AD pediatric patients

**FIGURE 1:**
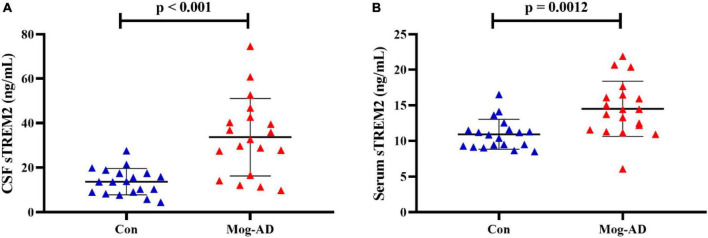
High level of CSF and serum sTREM2 in MOG-AD pediatric patients. **(A)** sTREM2 levels in cerebrospinal fluid samples from the pediatric MOG-AD group (*N* = 19) were significantly higher than the control groups (*N* = 19) (*p* < 0.001). **(B)** sTREM2 levels in serum samples from the pediatric MOD-AD group (*N* = 19) were significantly higher, compared with the control group (*N* = 19) (*p* = 0.0012). Independent two-sample *t*-test were used for differential analysis.

The CSF and serum sTREM2 levels are presented in Table 1. The median levels of CSF sTREM2 were 32.63 (9.76–74.61) ng/ml in the MOG-AD pediatric group and 13.69 (4.53–27.55) ng/ml in the control group. The median levels of serum sTREM2 were 14.42 (6.08–21.91) ng/ml in the MOG-AD pediatric group and 10.84 (8.49–14.5) ng/ml in the control group. The sTREM2 levels were significantly higher in the MOG-AD pediatric group than in the control group, both in CSF and serum (*p* < 0.001, Figure 1A, and *p* = 0.0012, Figure 1B, respectively).

### Cerebrospinal fluid soluble triggering receptor expressed on myeloid cells 2 levels concerning clinical inflammatory indexes and adapted modified Rankin Scale score

**FIGURE 2:**
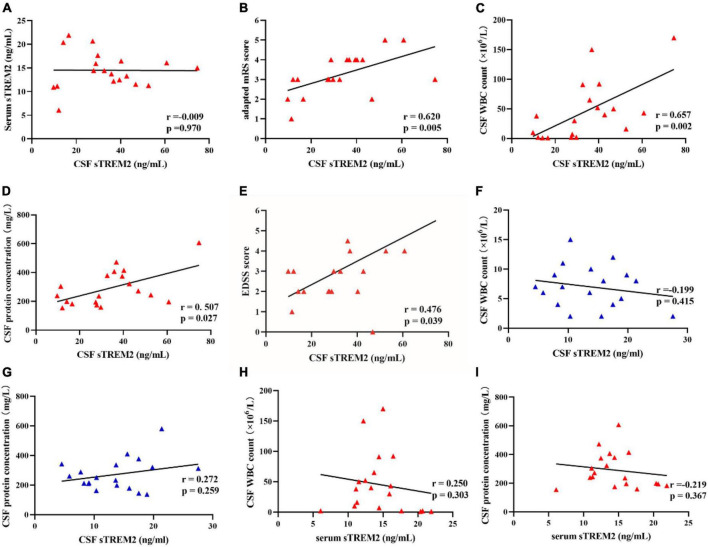
The CSF sTREM2 level was related to the adapted mRS score, EDSS score, and clinical inflammatory indexes. **(A)** There was no correlation between CSF sTREM2 levels and serum sTREM2 levels (Pearson correlation, *r* = –0.009, *p* = 0.970). **(B)** There was a mildly positive correlation between the adapted modified Rankin Scale (mRS) score and CSF sTREM2 levels (Spearman correlation, *r* = 0.620, *p* = 0.005). **(C,D)** There were significant correlations between CSF sTREM2 levels and central clinical inflammatory indexes. CSF sTREM2 levels were significantly positive correlated with the CSF WBC count (Spearman correlation, *r* = 0.657, *p* = 0.002). CSF sTREM2 levels were significantly positive correlated with CSF protein concentration (Spearman correlation, *r* = 0.507, *p* = 0.027). **(E)** The CSF sTREM2 levels were positively correlated to the Expanded Disability Status Scale (EDSS) score (Spearman correlation, *r* = 0.476, *p* = 0.039; **E**). **(F,G)** CSF sTREM2 levels had no correlation with the CSF WBC counts (Pearson correlation, *r* = –0.199, *p* = 0.415) and the CSF protein concentration (Pearson correlation, *r* = 0.272, *p* = 0.259) in control groups, respectively. **(H,I)** Serum sTREM2 levels had no correlation with the CSF WBC counts (Spearman correlation, *r* = –0.250, *p* = 0.303) and the CSF protein concentration (Spearman correlation, *r* = –0.219, *p* = 0.367) in pediatric MOG-AD groups, respectively.

Since there are no randomized controlled trials or consensus guidelines for the treatment of MOG-AD, we used an adapted modified Rankin Scale (mRS) to assess the disease severity (16). The CSF sTREM2 levels were mildly correlated with adapted mRS score (*r* = 0.620, *p* = 0.005; Figure 2B) and Expanded Disability Status Scale (EDSS) score (*r* = 0.476, *p* = 0.039; Figure 2E) in MOG-AD pediatric groups. Correlations between CSF sTREM2 levels and clinical inflammatory indexes were shown in Figures 2C,D. The CSF sTREM2 level was correlated with CSF biomarkers of inflammatory (CSF WBC count and CSF protein concentration). We discovered that, in MOG-AD pediatric group, CSF sTREM2 concentration were significantly associated with both CSF WBC count (*r* = 0.657, *p* = 0.002; Figure 2C) and CSF protein concentration (*r* = 0.507, *p* = 0.027; Figure 2D), while found no association described above in the control group (*r* = −0.199, *p* = 0.415, Figure 2F; *r* = 0.272, *p* = 0.259, Figure 2G; respectively). In this work, there is no correlation between patients’ age and CSF sTREM2 levels in MOG-AD group (data not shown).

However, the serum sTREM2 level showed no correlation with CSF WBC count (*r* = −0.25, *p* = −0.303, Figure 2H) in the pediatric MOG-AD group. There was no correlation between serum sTREM2 level with CSF protein concentration (*r* = −0.219, *p* = 0.367, Figure 2I) in the pediatric MOG-AD group, either. Besides, there was no association between the serum sTREM2 level with the CSF sTREM2 levels in the pediatric MOG-AD group (*r* = −0.009, *p* = 0.970, Figure 2A). These results indicated that sTREM2 had different sources in serum and CSF.

### The cerebrospinal fluid soluble triggering receptor expressed on myeloid cells 2 and serum soluble triggering receptor expressed on myeloid cells 2 levels can be a biomarker for MOG-AD diagnosis

**FIGURE 3:**
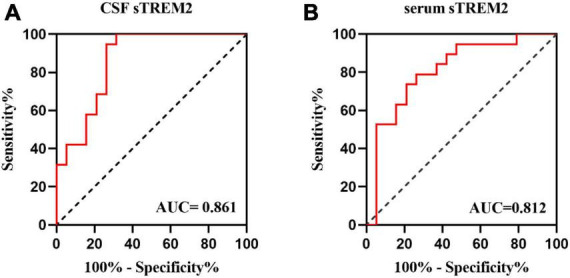
The CSF and serums TREM-2 levels can be a specific MOG-AD diagnosis biomarker. **(A,B)** Receiver operating characteristic (ROC) curve analysis of sTREM2 in CSF and serum for MOG-AD diagnosis (*N* = 38). **(A)** The area under the curve (AUC) of CSF sTREM2 levels was 0.861 (*p* < 0.001). **(B)** The area under the curve (AUC) of serum sTREM2 levels was 0.812 (*p* = 0.001).

To further investigate how well sTREM2 levels are capable of discriminating between pediatric MOG-AD patients and control groups, the ROC curve analysis was established. The CSF sTREM2 levels could differentiate pediatric MOG-AD patients (*N* = 19) well from the control group (*N* = 19), with the area under the curve (AUC) of CSF sTREM2 levels of 0.861 (*p* < 0.001; Figure 3A). The serum sTREM2 levels showed a slightly weaker result than the CSF sTREM2 levels, with an AUC of 0.812 (*p* = 0.001; Figure 3B).

## Discussion

The pathogenic mechanism of MOG-AD is still unclear. In the CNS, MOG locates on the external lamellae of myelin sheaths and the surface of the oligodendrocyte, which is associated with the myelination and the maturation of oligodendrocytes (17). There are a few types of research on the biological function of MOG. Experimental studies have revealed that MOG plays an essential role in maintaining the adhesion mechanisms of myelin (18) and interacting with the immune system. As the lesion of ADEM demonstrated (19), MOG-laden macrophages are found in the perivascular areas and demyelinating lesions. In this work, the higher level of CSF sTREM2 in the pediatric MOG-AD group further demonstrates that the activated microglia and macrophage may take part in the response to the MOG antibodies mediated neuroinflammation.

In this work, we revealed that: (1) the CSF and serum sTREM2 levels are higher in MOG-AD pediatric patients; (2) the CSF sTREM2 levels are correlated with CSF WBC count and CSF protein concentration; (3) the CSF sTREM2 levels are correlated with mRS score; and (4) the CSG sTREM2 levels could be a biomarker for MOG-AD diagnosis.

Besides, we also noted that there are no relationships between the serum sTREM2 levels with both CSF WBC count and CSF protein concentration. These negative results demonstrated that, compared with serum sTREM2 level, the CSF sTREM2 level has more reference value in measuring the degree of inflammation and may indicate the time phase of the inflammatory process.

As a soluble variant of TREM-2 detected in human biofluids (20), the differences in the sTREM2 levels in these biofluids might be a potential clue attributed to the microglial dysfunction and innate immunity. Although CSF sTREM2 levels have been studied extensively in neuroinflammatory diseases and neurodegeneration diseases, few studies have investigated the serum sTREM2 level. Several experimental studies have investigated that CSF sTREM2 levels were significantly elevated in Alzheimer’s disease (AD). It is strongly correlated with biomarkers of peripheral inflammation and the blood-brain barrier integrity biomarker (i.e., CSF Albumin/Serum Albumin ratio) (21). In MS, both CSF and serum sTREM2 level was higher in the MS group than in healthy control groups (22). By comparing the effects of natalizumab and mitoxantrone, only CSF sTRME2 has shown the potential utility as a biomarker for MS treatment effects (12). In addition, the level of CSF sTREM2 was increased in patients with neurosyphilis (NS), and peaked at the late stage, compared with the control groups (23).

To date, the clinical characteristics and prognosis of pediatric patients need more exploration. Pediatric patients had different clinical features: earlier age of onset, better recovery of visual acuity, lower annual rates of relapse, and more intracranial optic nerve involvement than middle-aged patients (3). The discovery of advanced antibodies refined our hypothesis about the immunopathogenesis of MOG-AD. MOG-AD is now recognized as an autonomous, antibody-mediated inflammatory demyelinating disorder. Thus, the approvement of MOG-AD diagnosis criteria requires more development in the pathophysiological mechanisms and the identification of markers. Our research is the first one investigating on the sTREM2 level in the pediatric MOG-AD patients’ biofluid and revealing the relationship between the CSF sTREM2 level with the clinical inflammatory indexes. TREM-2 is expressed on tissue macrophages in the peripheral and on microglia in the central (24). Research proved sTREM2 promotes microglial survival *via* PI3K/Akt pathway and activates the microglial depending on NF-κB (25).

We acknowledged a few limitations in this study, that is, lack of other neuroinflammatory disorders data, cross-sectional study design, single-center scope, and small sample size of Asians. In addition, as MOG-AD is rare, the sample of pediatric patients is limited. Since all the samples were collected before treatments, the treatment effects were not discussed in this study. It was difficult for pediatric MOG-AD patients to be accurately diagnosed and large treatment trials have not been performed (14).

## Conclusion

In conclusion, the higher level of CSF sTRME2 in pediatric MOG-AD patients supported the evidence that sTREM2 could be a marker of microglia activation in pediatric MOG-AD. These significant results indicated that sTREM2 could be useful in monitoring the degree of central inflammation.

## Data availability statement

The raw data supporting the conclusions of this article will be made available by the authors, without undue reservation.

## Ethics statement

The studies involving human participants were reviewed and approved by the Ethics Committee of Beijing Children’s Hospital Affiliated to Capital Medical University, Beijing, People’s Republic of China (Approved No. of the ethic committee: 2019-k-272). Written informed consent to participate in this study was provided by the participants’ legal guardian/next of kin. Written informed consent was obtained from the individual(s), and minor(s)’ legal guardian/next of kin, for the publication of any potentially identifiable images or data included in this article.

## Author contributions

AZ prepared the reagents, performed the experiments, and analyzed the data. CR and HC selected and clinically characterized the patients. JZ carried out statistical analyses. XR obtained the financial support. WZ designed the study. AZ wrote the manuscript with revisions from all authors.
